# Radiological phenotypes in pulmonary sarcoidosis: a reliability study of newly defined high-resolution computer tomography phenotypes

**DOI:** 10.1093/bjro/tzaf017

**Published:** 2025-06-25

**Authors:** Julie Van Woensel, Jasenko Krdzalic, Tom de Jaegere, Marlou T H F Janssen, Sofia Ramiro, César Magro Checa, Robert B M Landewé, Rémy L M Mostard

**Affiliations:** Department of Respiratory Medicine, Zuyderland Medical Centre, Heerlen, Limburg, 6419PC, The Netherlands; Department of Radiology, Zuyderland Medical Centre, Heerlen, Limburg, 6419PC, The Netherlands; Department of Radiology, Zuyderland Medical Centre, Heerlen, Limburg, 6419PC, The Netherlands; Department of Respiratory Medicine, Zuyderland Medical Centre, Heerlen, Limburg, 6419PC, The Netherlands; Department of Rheumatology, Zuyderland Medical Centre, Heerlen, Limburg, 6419PC, The Netherlands; Department of Rheumatology, Leiden University Medical Centre, Leiden, Zuid-Holland, 2311EZ, The Netherlands; Department of Rheumatology, Zuyderland Medical Centre, Heerlen, Limburg, 6419PC, The Netherlands; Department of Rheumatology, Zuyderland Medical Centre, Heerlen, Limburg, 6419PC, The Netherlands; Department of Rheumatology and Clinical Immunology, Amsterdam Rheumatology Centre, Amsterdam, Noord-Holland, AMC 1105AZ, The Netherlands; Department of Respiratory Medicine, Zuyderland Medical Centre, Heerlen, Limburg, 6419PC, The Netherlands; Department of Respiratory Medicine, Maastricht Universal Medical Centre, Maastricht, Limburg, 6229HX, The Netherlands

**Keywords:** pulmonary sarcoidosis, phenotyping, high resolution computer tomography, reliability

## Abstract

**Objectives:**

An accurate morphological classification of distinct pulmonary phenotypes in sarcoidosis is lacking. Recently, a multinational Delphi study was conducted to reach a consensus on recognizable high-resolution computer tomography (HRCT) phenotypes in pulmonary sarcoidosis as a basis for a more distinctive classification. The reliability of these phenotypes has not yet been evaluated.

**Methods:**

HRCT scans of adult sarcoidosis patients from the pulmonology department of a single sarcoidosis referral center were scored by three blinded independent readers. Seven phenotypes were distinguished as described in the Delphi study. They were divided into two subgroups: “non-fibrotic” and “likely-to-be fibrotic”. Intra- and inter-reader reliability for scoring the predominant phenotype and the subgroup was assessed using weighted Kappa (K_w_) statistics.

**Results:**

Forty-five patients (mean age, 47 years ± 12, 28 men) were included. For the scoring of the predominant phenotype, inter-reader reliability between all readers was substantial with an overall Fleiss’ kappa of 0.69 (CI 0.622-0.765, *P* < .001). We observed a substantial inter-reader reliability between readers A and B (*K*_w_ of 0.76), between readers B and C (K_w_ of 0.66) and between readers A and C (*K*_w_ of 0.66). For the scoring of the subgroups “non-fibrotic” vs. “likely-to-be fibrotic,” overall Fleiss’ Kappa was substantial (*K* = 0.78, CI 0.607-0.944, *P* < .001). We observed a *K*_w_ score of 0.76 between readers A and B; 0.81 between readers A and C; 0.76 between readers B and C. Intra-reader reliability was substantial between the scores of A in scoring the predominant phenotypes (*K*_w_ of 0.71) and it was almost perfect in scoring the subgroups (*K*_w_ of 0.95). Intra-reader reliability was substantial between the scores of B in scoring the predominant phenotype (*K*_w_ of 0.66) and subgroups (*K*_w_ of 0.72).

**Conclusions:**

The inter- and intra-reader reliability of the newly proposed HRCT phenotypes obtained from the Delphi study is very acceptable.

**Advances in knowledge:**

This study is the first to assess the reliability of these HRCT phenotypes and supports the use of them for classification purposes in future clinical and pathogenetic studies.

## Introduction

Sarcoidosis is a multisystemic disease with a highly variable clinical presentation, natural course, and prognosis.^1^ Pulmonary involvement is present in more than 90% of newly diagnosed sarcoidosis patients.^2^ Pulmonary manifestations in patients can be highly variable: asymptomatic cases and cases with spontaneous remission are frequently seen, but cases with chronic progressive disease and cases with pulmonary fibrosis with high mortality may also occur.^3^ Airway as well as interstitial involvement can be present, resulting in different imaging and functional phenotypes, which are associated with variable disease outcomes.^4^^,^^5^

The radiographic staging described by Scadding,^6^ based on a chest radiograph (CXR), is not sensitive enough to show early parenchymal involvement and detailed morphological patterns. Moreover, its use is limited by inter- and intra-reader variability.^7^ The CXR staging is roughly associated with pulmonary outcome,^6^ but a clear relationship between these radiographic stages and lung function parameters or disease activity is absent.^3^^,^^8^

Imaging by high-resolution computer tomography (HRCT) is superior to CXR in detecting limited parenchymal involvement and details such as nodules, (non-)septal lines, and irregularity of the bronchovascular bundle.^5^ Furthermore, HRCT has been demonstrated to be helpful in the discrimination between reversible lesions with possible active inflammation and irreversible fibrosis.^9–11^ Several studies also showed that some HRCT abnormalities are associated with patterns of lung function impairment and disease severity.^12–14^

An accurate morphological HRCT classification could contribute to therapeutic decision-making and estimating prognosis, but such classification is lacking. In the past, studies have been conducted to investigate HRCT scores in pulmonary sarcoidosis, but their scoring methods were individually defined rather than established by international expert consensus and HRCT techniques were less developed.^12^^,^^14–17^

**Figure 1.**
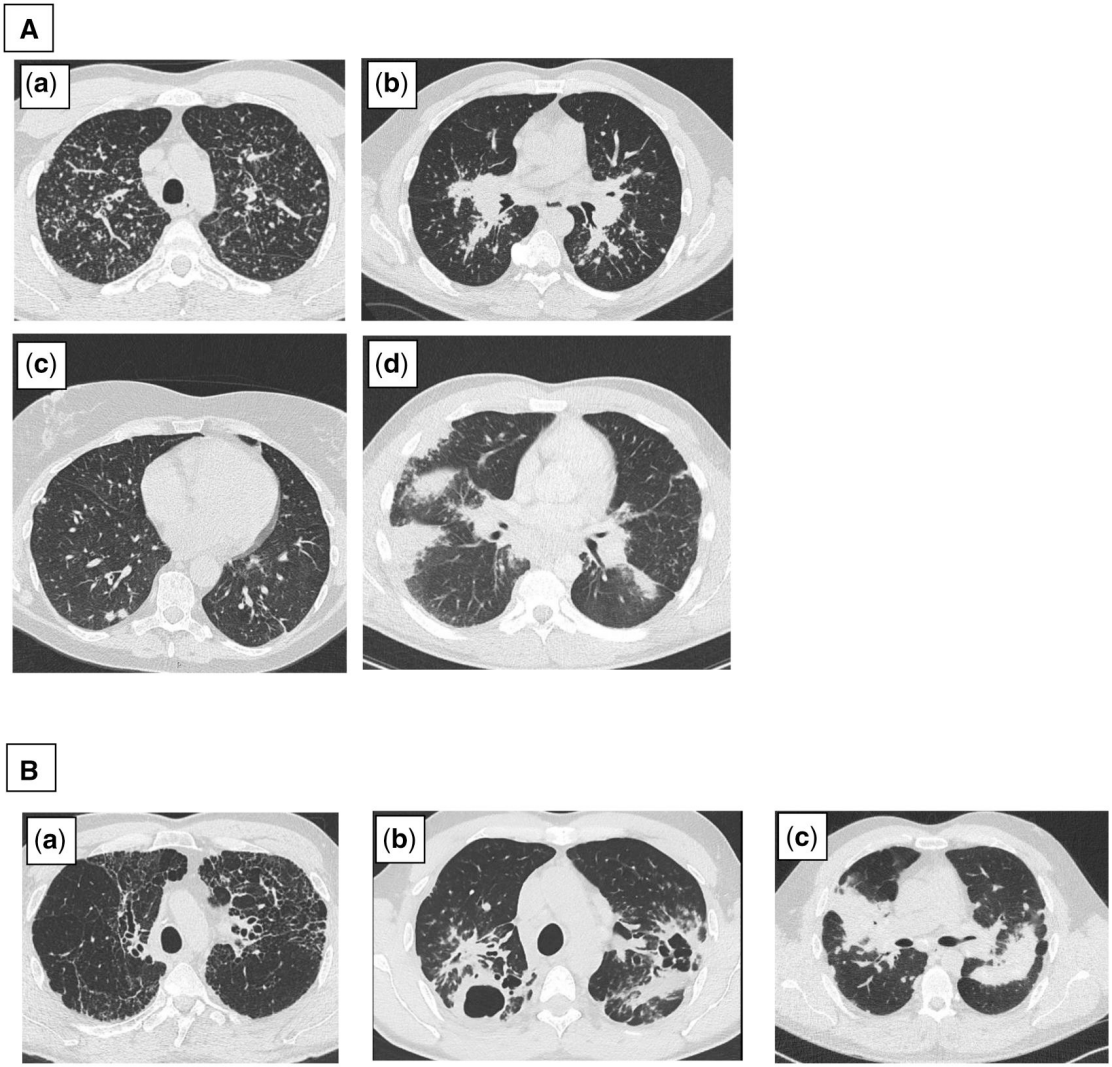
HRCT phenotypes from the Delphi study. (**A**): “non-fibrotic” phenotypes: (a) Phenotype 1, Multiple peri-bronchovascular, peri-fissural, or subpleural micronodules. (b) Phenotype 2: Multiple larger peri-bronchovascular nodules; (c) Phenotype 3: Scattered larger nodules; (d) Phenotype 4: Consolidation as the predominant or sole abnormality; (**B**): “Likely fibrotic” phenotypes: (a) Phenotype 5: Bronchocentric reticulation with or without dense parenchymal opacification, without cavitation; (b) Phenotype 6: Bronchocentric reticulation and dense parenchymal opacification, with cavitation; (c) Phenotype 7: Large bronchocentric masses ie progressive massive fibrosis (PMF) lookalike.

Using a Delphi study, a recent international consensus was reached on newly recognizable HRCT phenotypes in pulmonary sarcoidosis (Figure 1).^18^ The expert panel reached a consensus on seven distinct HRCT phenotypes and categorized them into two subgroups describing “non-fibrotic” and “likely fibrotic” phenotypes. Those phenotypes have not yet been further assessed, which means that there is no information available on their predictive validity nor on the reliability of their assessment.

The aim of our study was to assess the inter- and intra-reader reliability of these newly proposed HRCT phenotypes in pulmonary sarcoidosis patients. To the best of our knowledge, this is the first validation study of these HRCT phenotypes.

## Methods

### Study design and subjects

This cross-sectional retrospective study was conducted in a Dutch cohort of adult patients with sarcoidosis from the pulmonology department of a large regional referral center for sarcoidosis in the Netherlands. The study was approved by the local medical ethical committee (METCZ20230115) and performed in compliance with the guidelines for reporting reliability and agreement studies (GRRAS).^20^

All patients had an established diagnosis of sarcoidosis in accordance with the consensus statement on sarcoidosis of the American Thoracic Society/European Respiratory Society/World Association of Sarcoidosis and Other Granulomatous Disorders.^1^

To avoid unreliable kappa scores, we selected a balanced number of CT scans per phenotype and in line with that the total sample size of a minimal of 42 CT scans. Out of a sample of baseline and follow-up scans, the scans were selected by a pulmonologist with expertise in interpreting sarcoidosis HRCT, based on the phenotypes out of the Delphi study (Figure 1).^18^

Exclusion criteria were the presence of other pulmonary diseases interfering with radiographic features (eg, emphysema or acute pulmonary infections) and low-quality CT images.

### Procedures and data collection

#### Chest CT-protocol

Chest CT scans were performed on either a 16-slice (Siemens Sensation 16) or 64-slice (Siemens Somatom Definition AS) CT scanner or on a 64-slice dual source scanner (Siemens Somatom Definition Flash). Patients were scanned in the supine position during breath holding, at a tube voltage pre-set of 120 kilovoltage (kV), with automated modulating milliamperes-seconds (mAs), at slice thickness of 0.6-1.0 mm. CT images were reconstructed in the transverse, coronal and sagittal planes. One scan extracted from another center was performed with a different scanner but with identical scanning parameters. Six scans (13%) had a coupe-thickness of more than 1 mm because the initial clinical indication for those scans was suspicion of malignancy or pulmonary embolism. Images were available in the local picture archiving and communication system (Sectra IDS7 PACS).

#### The reading procedure of CT scans

Pulmonary involvement was classified according to the new HRCT phenotypes from the Delphi study (Figure 1).^18^ Seven phenotypes were distinguished; Phenotype 1: multiple peri-bronchovascular, peri-fissural, or subpleural micronodules; Phenotype 2: multiple larger peri-bronchovascular nodules; Phenotype 3: scattered larger nodules; Phenotype 4: consolidation as the predominant or sole abnormality; Phenotype 5: bronchocentric reticulation with or without dense parenchymal opacification, without cavitation; Phenotype 6: bronchocentric reticulation and dense parenchymal opacification, with cavitation; Phenotype 7: large bronchocentric masses ie progressive massive fibrosis (PMF) lookalike. The phenotypes were divided into two subgroups: “non-fibrotic” (phenotypes 1-4) and “likely-to-be fibrotic” (phenotypes 5-7). Each reader identified the most prominent phenotype. Intra- and inter-reader reliability was assessed for the evaluation of the predominant phenotype (1-7) and the subgroup (“non-fibrotic” vs. “likely-to-be fibrotic”).

To assess inter-reader reliability, the scans were separately scored by three readers (reader A [JvW]; reader B [JK]; reader C [TdJ]). Each reader was blinded tot the scoring of the selector (RM), the scoring of the other readers and to any clinical information. Before the study, the readers underwent a training session to ensure that they understood the description of each of the phenotypes. This training was performed by means of random patient samples. To assess intra-reader reliability, reader A and reader B scored the CT images twice, with an interval of 6 months.

### Statistical analysis

A proportionate presence with at least five scans per phenotype was intended for. Weighted kappa (*K*_w_) statistics were used to assess the inter- and intra-reader reliability of these categorical data. Fleiss’ Kappa (K) statistics were used to assess overall reliability for more than two readers. Kappa values were interpreted as: <0 poor agreement, 0.00-0.20 slight agreement, 0.21-0.40 fair agreement, 0.41-0.60 moderate agreement, 0.61-0.80 substantial agreement, 0.81-1.00 almost perfect agreement.^21^ Data were analyzed with SPSS Statistics version 29.

## Results

### Subjects

A total of 45 patients were included in this study. Of these, 62% were male, and the mean age at the time of CT scanning was 47 years. All demographic characteristics and radiologic stages by Scadding are listed in Table 1.

**Table 1.** Demographic and clinical characteristics.

Variable	*N* = 45
Age at time of CT-scan—years^a^	47 (12)
*Sex* ^b^	
Male	28 (62)
*Ethnicity* ^b^	
White	40 (89)
Black	2 (4)
Asian	0 (0)
Other	3 (7)
*Smoking status* ^b^	
Active	2 (4)
Former	18 (40)
Never	25 (56)
*Initial presentation* ^b^	
Incidental diagnosis	2 (4)
Symptomatic	43 (96)
Histological diagnosis^b^	37 (86)
Radiographic stage by X-ray (Scadding) ^b, c^ Stage 0 Stage 1 Stage 2 Stage 3 Stage 4	4 (9) 2 (5) 14 (32) 15 (35) 8 (19)

Data were missing for the following variables: histology (2 patients, <5%), radiologic stage (2 patients, <5%).

Abbreviation: CT = computer tomography.

a Variables are presented as mean (standard deviation).

b Variables are presented as numbers (percentages).

c Radiographic staging by Scadding (9): Stage 0, normal X-ray; stage 1, bilateral hilar lymphadenopathy; stage 2, bilateral hilar lymphadenopathy and parenchymal abnormality; stage 3, parenchymal abnormality without bilateral hilar lymphadenopathy; stage 4, advanced pulmonary fibrosis.

### Distribution of CT phenotypes

The frequency of each HRCT phenotype according to the selector’s scoring was as follows: phenotype 1 7/45 (15%), phenotype 2 5/45 (15%), phenotype 3 8/45 (13%), phenotype 4 5/45 (11%), phenotype 5 8/45 (18%), phenotype 6 4/45 (9%), phenotype 7 8/45 (18%).

### Inter-reader reliability

For the scoring of the predominant phenotype, inter-reader reliability between all readers was substantial with an overall Fleiss’ kappa of 0.69 (CI 0.622-0.765, *P* < .001). We observed a K_w_ score of 0.76 between readers A and B; 0.66 between readers A and C; 0.66 between readers B and C (Table 2).

**Table 2.** Inter-reader agreement for recognizing the predominant HRCT pattern in pulmonary sarcoidosis patients.

Reader A	Reader B							
	P1	P2	P3	P4	P5	P6	P7	Total
P1	3	0	0	0	2	0	0	5
P2	0	7	0	0	0	0	0	7
P3	1	0	7	0	0	0	0	8
P4	0	1	0	6	0	0	1	8
P5	0	0	0	0	7	0	0	7
P6	0	0	0	0	1	3	0	4
P7	0	1	0	3	0	0	3	6
Total	4	8	7	9	10	3	4	45
*K*_w_	0.76							

Reader A	Reader C							
	P1	P2	P3	P4	P5	P6	P7	Total
P1	3	1	0	0	1	0	0	5
P2	0	5	1	1	0	0	0	7
P3	1	0	6	1	0	0	0	8
P4	0	2	0	5	0	0	1	8
P5	0	0	0	1	5	1	0	7
P6	0	0	0	0	0	4	0	4
P7	0	1	0	1	0	0	4	6
Total	4	9	7	9	6	5	5	45
*K*_w_	0.66							

Reader B	Reader C							
	P1	P2	P3	P4	P5	P6	P7	Total
P1	3	1	0	0	0	0	0	4
P2	0	6	1	1	0	0	0	8
P3	0	0	6	1	0	0	0	7
P4	0	2	0	5	0	0	2	9
P5	1	0	0	1	6	2	0	10
P6	0	0	0	0	0	3	0	3
P7	0	0	0	1	0	0	3	4
Total	4	9	7	9	6	5	5	45
*K*_w_	0.66							

Tables refer to absolute numbers.

Abbreviations: Kw = Weighed Kappa score; P1 = Phenotype 1, Multiple peri-bronchovascular, peri-fissural, or subpleural micronodules; P2 = Phenotype 2, Multiple larger peri-bronchovascular nodules; P3 = Phenotype 3, Scattered larger nodules; P4 = Phenotype 4, Consolidation as the predominant or sole abnormality; P5 = Phenotype 5, Bronchocentric reticulation with or without dense parenchymal opacification, without cavitation; P6 = Phenotype 6, Bronchocentric reticulation and dense parenchymal opacification, with cavitation; P7 = Phenotype 7, Large bronchocentric masses.

Detailed weighted kappa scores between each pair of readers for all individual phenotypes are shown in Supplementary Tables S1-S3. Table 3 shows the overall reliability for all individual phenotypes between all three readers calculated with Fleiss’ kappa. The phenotypes with the highest agreement between readers were phenotypes 3, 5, and 6, while those with the lowest agreement between readers were phenotypes 2, 4, and 7.

**Table 3.** Overall inter-reader agreement with Fleiss’ kappa scores.

Subjects	P1	P2	P3	P4	P5	P6	P7
1				3			
2		3					
3					3		
4	2	1					
5							
6							
7				1			2
8				1			2
9		3					
10		2	1				
11			3				
12			2	1			
13				1	2		
14							3
15	2				1		
16	1				2		
17					3		
18				3			
19			3				
20	3						
21			3				
22					2	1	
23		1		1			1
24						3	
25				1			2
26				3			
27				3			
28				3			
29				3			
30	2		1				
31		2		1			
32					3		
33		3					
34			3				
35			3				
36							3
37					3		
38					1	2	
39		3					
40	3						
41				1			2
42				3			
43						3	
44		3					
45		3					
*K*	0.66	0.70	0.84	0.52	0.74	0.82	0.63
*K*_overall_	0.69 (CI 0.622-0.765, *P* < .001)						

Abbreviations: CI = confidence interval; K = Fleiss’ Kappa score; P1 = Phenotype 1, Multiple peri-bronchovascular, peri-fissural, or subpleural micronodules; P2 = Phenotype 2, Multiple larger peri-bronchovascular nodules; P3 = Phenotype 3, Scattered larger nodules; P4 = Phenotype 4, Consolidation as the predominant or sole abnormality; P5 = Phenotype 5, Bronchocentric reticulation with or without dense parenchymal opacification, without cavitation; P6 = Phenotype 6, Bronchocentric reticulation and dense parenchymal opacification, with cavitation; P7 = Phenotype 7, Large bronchocentric masses.

For the scoring of the subgroups “non-fibrotic” vs. “likely-to-be fibrotic,” overall Fleiss’ Kappa was substantial (*K* = 0.78, CI 0.607-0.944, *P* < .001). We observed a *K*_w_ score of 0.76 between readers A and B; 0.81 between readers A and C; 0.76 between readers B and C.

#### Intra-reader reliability

Intra-reader reliability was substantial between the scores of reader A in scoring the predominant phenotypes (*K*_w_ of 0.71) and it was almost perfect in scoring the subgroups (*K*_w_ of 0.95). Intra-reader reliability was substantial between the scores of reader B in scoring the predominant phenotype (*K*_w_ of 0.66) and subgroups (*K*_w_ of 0.72).

## Discussion

In this study, we assessed the inter-reader and intra-reader reliability of newly defined HRCT phenotypes, from a recent international Delphi study, in a Dutch population with pulmonary sarcoidosis. We found substantial inter- and intra-reader reliability, both for the scoring of the predominant phenotype and for the classification into “non-fibrotic” vs. “likely to be fibrotic” subgroups.

An interesting characteristic of this HRCT phenotype distinction is its morphological connotation, namely the division between “possibly non-fibrotic” and “likely fibrotic” phenotypes.

The different HRCT phenotypes from the Delphi study are classified according to a possible underlying pathophysiology in pulmonary sarcoidosis, making it particularly relevant for future clinical and pathogenetic studies. This is in contrast with previous proposed HRCT scoring systems in this disease, which categorized findings solely based on the type of CT abnormalities (eg, ground-glass, nodules, reticulation, honeycombing).^13^^,^^15–17^^,^^22^ Other studies investigated the relationship between specific HRCT phenotypes and pulmonary function, but these studies focused only on the fibrotic subtypes or airway involvement.^12^^,^^20^^,^^23^ By subdividing phenotypes into “non-fibrotic” and “likely fibrotic” phenotypes, the investigators of the Delphi study suggest a potential role for HRCT in the differentiation between inflammatory and fibrotic disease in pulmonary sarcoidosis, supported by previous data.^9–11^ In our study, we observed strong agreement when assessing these two subgroups, indicating the potential clinical value of this phenotypic distinction in future clinical studies. The goal of the Delphi study was to define CT phenotypes that better correlate with disease course. Peri-lymphatic nodules and peri-bronchovascular nodules, consolidation, for masses are suspected to be reversible either spontaneously or under anti-inflammatory treatment, while fibrotic patterns with extensive reticulation and cavitation are suspected to be irreversible. However, in pulmonary sarcoidosis, even “likely fibrotic” radiological patterns can still show inflammatory activity on a positron emission tomography with 2-deoxy-2-[fluorine-18] fluoro-d-glucose integrated with computed tomography (^18^F-FDG PET/CT) (11). Future clinical studies are warranted to explore the correlation between imaging, inflammatory, and functional parameters to better predict disease course and guide treatment decision.

Another important advantage of this phenotype classification is that it is a qualitative method, in contrast to the more time-consuming (semi-)quantitative methods that have been used in previous studies.^9^^,^^13^^,^^15^^,^^16^^,^^22^ By scoring the predominant phenotype, a clear classification can be made in a fast manner that is feasible for future research.

While our study demonstrates the reliability of this phenotypic classification, we have also experienced some of its limitations. First, the radiological patterns per phenotype were not described in sufficient detail, reflecting the lower agreement between readers on phenotypes 2, 4, and 7. Phenotype 7 (large bronchocentric masses) was difficult to distinguish from phenotype 2 (larger peri-bronchovascular nodules) and phenotype 4 (predominant consolidations); Larger peri-bronchovascular nodules that confluence can resemble bronchocentric masses; Consolidations that are more centrally localized or bronchocentric masses that extend into the peripheral zones are difficult to discriminate. Because of the summary description, a reader could give its own interpretation to the phenotypes, possibly reducing both inter- and intra-reader agreement. We suggest that the terms “air bronchogram” for consolidations and “architectural distortion” and “volume loss” for the “likely fibrotic” patterns could be added to the description of the phenotypes. Furthermore, we experienced that it is difficult in some cases to indicate a predominant phenotype when more phenotypes are simultaneously present.

Our study has more limitations. We recognize the limited sample size of our study. We can argue this sample size, however. Certain phenotypes (such as 4, 6, and 7) in pulmonary sarcoidosis are less frequent. Augmenting our sample size would disproportionally increase other phenotypes (1, 2, and 3) with a risk of unreliable high kappa scores.

Secondly, six out of 45 scans had a coupe thickness of >1 mm; however, the image quality was considered sufficient to score because the predominant pattern in those scans was pattern 3 or 4, and thin slices are not strictly required to distinct these patterns.

The strength of our study is that it was conducted according to the GRRAS guideline. Furthermore, the CT scores were based upon a large international consensus obtained by a standardized Delphi study. The CT scans were scored by more than two readers, and that the readers were trained prior to the study.

Based on our findings, further external validation across multiple centers with a larger patient population would enhance the generalizability of our findings. Future studies are also planned to investigate the clinical utility, causal relationship and treatment response of this new CT phenotypes.

## Conclusion

In this first validation study of newly defined HRCT phenotypes from the Delphi study in a Dutch population with pulmonary sarcoidosis, we found a substantial inter- and intra-reader reliability. Our results support the use of this system for further studies to evaluate the clinical value of these new HRCT phenotypes by ensuring the possibility to assess them reliably.

## Supplementary Material

tzaf017_Supplementary_Data
