# Supplementary material for: Radiological phenotypes in pulmonary sarcoidosis: a reliability study of newly defined high-resolution computer tomography phenotypes
Source: BJR Open. 2025 Jun 25;7(1):tzaf017. doi: 10.1093/bjro/tzaf017 (PMC12255234; doi:10.1093/bjro/tzaf017)
Supplement: tzaf017_Supplementary_Data [file tzaf017_supplementary_data.docx]

| **Table S1.** Inter-reader reliability between reader A and B for each individual phenotype | | | | | | | | |
| --- | --- | --- | --- | --- | --- | --- | --- | --- |
|  | |  |  |  |  |  |  |  |
| Reader A | Reader B | | | | | | | |
|  | No. positive | | | | No. negative | | | |
| P 1  No. positive  No. negative  % agreement  K_w_ | 3  1  75  0.63 | | | | 2  39  95 | | | |
| P 2  No. positive  No. negative  % agreement  K_w_ | 7  1  87  0.92 | | | | 0  37  100 | | | |
| P 3  No. positive  No. negative  % agreement  K_w_ | 7  0  100  0.92 | | | | 1  37  97 | | | |
| P 4  No. positive  No. negative  % agreement  K_w_ | 6  3  67  0.64 | | | | 2  34  94 | | | |
| P 5  No. positive  No. negative  % agreement  K_w_ | 7  3  70  0.78 | | | | 0  35  100 | | | |
| P 6  No. positive  No. negative  % agreement  K_w_ | 3  0  100  0.85 | | | | 1  41  98 | | | |
| P 7  No. positive  No. negative  % agreement  K_w_ | 3  1  75  0.55 | | | | 3  38  93 | | | |

Abbreviations : Kw : Weighed Kappa score; P1: Phenotype 1, Multiple peri-bronchovascular, peri-fissural, or subpleural micronodules; P2: Phenotype 2, Multiple larger peri-bronchovascular nodules; P3: Phenotype 3, Scattered larger nodules; P4 : Phenotype 4, Consolidation as the predominant or sole abnormality; P5: Phenotype 5, Bronchocentric reticulation with or without dense parenchymal opacification, without cavitation; P6: Phenotype 6 , Bronchocentric reticulation and dense parenchymal opacification, with cavitation; P7: Phenotype 7, Large bronchocentric masses

| **Table S2.** Inter-reader agreement between reader A and C for each individual phenotype | | | | | | | | |
| --- | --- | --- | --- | --- | --- | --- | --- | --- |
|  | |  |  |  |  |  |  |  |
| Reader A | Reader C | | | | | | | |
|  | No. positive | | | | No. negative | | | |
| P 1  No. positive  No. negative  % agreement  K_w_ | 3  1  75  0.63 | | | | 2  39  95 | | | |
| P 2  No. positive  No. negative  % agreement  K_w_ | 5  4  56  0.54 | | | | 2  34  94 | | | |
| P 3  No. positive  No. negative  % agreement  K_w_ | 6  1  86  0.76 | | | | 2  36  95 | | | |
| P 4  No. positive  No. negative  % agreement  K_w_ | 5  4  56  0.49 | | | | 3  33  92 | | | |
| P 5  No. positive  No. negative  % agreement  K_w_ | 5  1  83  0.73 | | | | 2  37  95 | | | |
| P 6  No. positive  No. negative  % agreement  K_w_ | 4  1  80  0.88 | | | | 0  40  100 | | | |
| P 7  No. positive  No. negative  % agreement  K_w_ | 3  1  75  0.55 | | | | 3  38  93 | | | |

Abbreviations : Kw : Weighed Kappa score; P1: Phenotype 1, Multiple peri-bronchovascular, peri-fissural, or subpleural micronodules; P2: Phenotype 2, Multiple larger peri-bronchovascular nodules; P3: Phenotype 3, Scattered larger nodules; P4 : Phenotype 4, Consolidation as the predominant or sole abnormality; P5: Phenotype 5, Bronchocentric reticulation with or without dense parenchymal opacification, without cavitation; P6: Phenotype 6 , Bronchocentric reticulation and dense parenchymal opacification, with cavitation; P7: Phenotype 7, Large bronchocentric masses (ie, PMF lookalike)

| **Table S3.** Inter-reader agreement between reader B and C for each individual phenotype | | | | | | | | |
| --- | --- | --- | --- | --- | --- | --- | --- | --- |
|  | |  |  |  |  |  |  |  |
| Reader B | Reader C | | | | | | | |
|  | No. positive | | | | No. negative | | | |
| P 1  No. positive  No. negative  % agreement  K_w_ | 3  1  75  0.73 | | | | 1  40  98 | | | |
| P 2  No. positive  No. negative  % agreement  K_w_ | 6  3  67  0.64 | | | | 2  34  94 | | | |
| P 3  No. positive  No. negative  % agreement  K_w_ | 6  1  86  0.83 | | | | 1  37  97 | | | |
| P 4  No. positive  No. negative  % agreement  K_w_ | 5  4  56  0.44 | | | | 4  32  89 | | | |
| P 5  No. positive  No. negative  % agreement  K_w_ | 6  0  100  0.70 | | | | 4  35  90 | | | |
| P 6  No. positive  No. negative  % agreement  K_w_ | 3  2  60  0.73 | | | | 0  40  100 | | | |
| P 7  No. positive  No. negative  % agreement  K_w_ | 2  2  50  0.45 | | | | 2  39  95 | | | |

Abbreviations : Kw : Weighed Kappa score; P1: Phenotype 1, Multiple peri-bronchovascular, peri-fissural, or subpleural micronodules; P2: Phenotype 2, Multiple larger peri-bronchovascular nodules; P3: Phenotype 3, Scattered larger nodules; P4 : Phenotype 4, Consolidation as the predominant or sole abnormality; P5: Phenotype 5, Bronchocentric reticulation with or without dense parenchymal opacification, without cavitation; P6: Phenotype 6 , Bronchocentric reticulation and dense parenchymal opacification, with cavitation; P7: Phenotype 7, Large bronchocentric masses (ie, PMF lookalike)
